# Receipt of prescription opioid medication is associated with increased mortality in an Israeli population

**DOI:** 10.1186/s13584-024-00606-y

**Published:** 2024-04-03

**Authors:** Matan J. Cohen, Reuven L. Dressler, Ehud Kaliner

**Affiliations:** 1 Clalit Health Services, Jerusalem district, Hebrew University of Jerusalem Faculty of Medicine, Bet Shemesh, Israel; 2 Clalit Health Services, Department of Family Medicine, Jerusalem district, Hebrew University of Jerusalem Faculty of Medicine, Maale Adumim, Israel; 3 State of Israel Ministry of Health, Central District, Ramla, Israel

**Keywords:** Opioid use, Prescription medicine addiction, Mortality

## Abstract

**Background:**

Despite Israel’s increased use of prescription opioids, reported deaths resulting or associated with opioids have decreased, in fact dramatically, since 2005. This contrast is unique and difficult to explain. We sought to examine whether higher prescribed opioid dosages among adults without oncologic diagnoses were associated with higher all-cause mortality rates.

**Methods:**

A historical cohort study in Clalit Health Services, using a data repository including all adult patients prescribed opiates between 2010 and 2020, excluding patients with oncologic diagnoses. Patients were classified into three groups according to opioid use: below 50 Morphine milligram equivalents (MME) per day, 50 to 90 MME per day, and above 90 MME per day. Sex, Charlson comorbidity score, age and socioeconomic status were recorded. Mortality rates were compared between the dosage groups and compared to age-standardized mortality rates in the general population.

**Results:**

On multivariate analysis, patients receiving 90 or more MME per day were 2.37 (95%CI 2.1 to 2.68) more likely to have died compared to patients receiving below 50 MME per day. The respective hazard ratio among patients receiving between 50 and 90 MME per day was 2.23 (2.01 to 2.46). Among patients aged 18 to 50, standardized mortality ratios (SMRs) compared to the general population ranged between 5.4 to 8.6 among women, receiving between 50 and 90 MME per day, and between 8.07 and 10.7 among women receiving 90 or more MME per day. The respective SMRs among men were 1.2 to 3.8 and 2.7 to 5.4.

**Conclusion:**

Increased opioid use is independently associated with increased all-cause mortality among non-oncological patients. This result is most notable among young adults with little or no known comorbidities. These findings are consistent with results in other countries and seem more credible than previous Israeli reports. Healthcare regulators and providers should, therefore, act to curtail the increasing opioid prescriptions and devise and enhance controls in the healthcare system, which, until 2020, had very limited mechanisms in place.

## Introduction

Israel has seen a consistent rise of opioid use between 2000 to 2021 [1–4], with trends similar to those reported in North America, the UK and Europe and of a magnitude as pronounced as the USA [5–8]. Notably, prescription opioids are increasingly used among Israeli patients who do not have oncologic diagnoses, and among these patients, the amount of fentanyl and oxycodone prescription use has increased in at least 700% since 2008 [9].

Along with increased opioid prescribing, increases in opioid-associated mortality were observed in many nations, most particularly in the United States [8, 10–12]. Despite Israel’s increased use of prescription opioids, reported deaths resulting or associated with opioids have decreased, in fact dramatically, since 2005, based on mortality certificates [13, 14]. This contrast is unique and difficult to explain.

One of the challenges in assessing the association between mortality and specific exposures, such as opioid medications, is the potential confounding effect comorbidities have. Comorbidities are associated with increased mortality and increased co-morbidities might and do inflict pain and suffering which are often alleviated with opioid medications. The Charlson comorbidity score is a well-validated and widely used proxy measure of the risk of death [15–18]. This score can, therefore, serve as a controlling measure to examine the existence of an independent association between opioid use and mortality.

We sought to examine all-cause mortality rates among adults without oncologic diagnoses who were prescribed opioids using a large data repository that contains just over one million patients prescribed opioids over a decade. We hypothesized that in this large cohort, we could assess whether opioid prescription, as measured by morphine milliequivalents (MME), was associated with higher mortality, especially by examining young healthy adults with no or few comorbidities.

## Methods

We conducted a historical cohort analysis using a data repository including all adult patients in Clalit Health Services (CHS) prescribed opioids between 2010 and 2020, excluding patients with oncologic diagnoses. We assessed whether there exists an association between total opioid prescription filled and all-cause mortality, as detailed below.

The data presented in this report were derived from CHS using the Clalit Research Data sharing platform powered by MDClone (https://wwwmdclone.com). CHS is the largest healthcare provider in Israel, serving above 51% of the population, totaling about 5 million individuals [19]. After obtaining authorization from the CHS committee for the protection of human research subjects, all CHS members above 18 years of age who had at least one opioid prescription filled between 2010 and 2020 and who were alive after December 31st 2010 ﻿were included in the analysis. Mortality was assessed between January 1st, 2011 and December 31st, 2020 (thereby assessing the effect of 2010 prescription only on patients who were alive in 2011 and onwards). Opioids were classified according to ATC level 5 opioid ingredients (buprenorphine, fentanyl, morphine, oxycodone and tramadol). Codeine was excluded, as its use was both common and stable with no effect on larger trends. Propoxyphene and pethidine were prescribed infrequently and also excluded. Medication-assisted treatment with methadone and buprenorphine (oral and sublingual) are not prescribed in the community-based healthcare system in CHS, but rather only in designated rehabilitation clinics (either government-run or privately licensed) and as such were not included in our analysis.

Morphine milligram equivalents (MME) for each filled prescription were calculated according to accepted methods [20]. Patients’ total filled MME was divided by the length of follow-up to derive the average MME per day. Patients were classified into three groups according to opioid use: below 50 MME per day, 50 to 90 MME per day, and above 90 MME per day, according to conventions used in the landmark 2016 CDC guideline [21].

Patient characteristics included in this analysis are: sex (female/male), age in 2010, Socioeconomic status (SES) stratum (very low / low / medium / high / very high, according to address-based designations of the Israeli Central Bureau of Statistics), Charlson comorbidity score [15], filled benzodiazepine prescriptions (yes/no), filled stimulant prescriptions (yes/no), year of death. Patients diagnosed with solid malignancy, leukemia or lymphoma were classified as oncology patients and excluded from the analysis.

Patient characteristics were compared between the three MME filling groups. Age was compared using the analysis of variance test, and all other categorical variables were compared using the chi-square test.

Mortality was assessed by generating a multivariate model. We employed Cox regression methods to estimate hazard ratios of the collected variables. In this model, the time-to-event was measured from the first prescription of opioid medication to death or censorship (the end of the study period). Additionally, in another analysis, we performed indirect standardization and calculated standardized mortality ratios (SMRs) by dividing observed 10-year mortality counts by the respective expected mortality counts, thereby controlling for age distribution and sex. The expected mortality rates per age and sex are published by the Israeli Central Bureau of Statistics [22]. SMR values above 1 indicated observed mortality counts higher than expected. Suspecting opioids to be associated with excess mortality among young and healthy individuals, we limited our SMR calculation to patients aged between 18 and 50 in 2010, whose Charlson comorbidity index was between zero and 10. We considered two sided *p*-values below 0.05 to be statistically significant. SPSS was used for all statistical analysis (IBM Corp. Released 2020. IBM SPSS Statistics for Windows, Version 27.0. Armonk, NY: IBM Corp).

## Results

There were 1,008,125 patients who received opioids between 2010 and 2020 and were alive on January 1, 2011. There were 1,001,719 patients who received on average less than 50 MME per day, 3606 received between 50 and 90 MME per day and 2800 received 90 or more MME per day. Non-oncological patients comprised 820,131 of the total cohort (81.3%); of these 817,097 (99.6%) received less than 50 MME per day 1778 (0.2%) received on average between 50 and 90 MME per day, and 1256 (0.15%) received on average 90 or more MME per day.

Greater MME filling was more common among males, in the very low and low SES strata, and was associated more often with benzodiazepine and stimulant filling. Patients’ age was similar between the groups (Table 1). Among patients receiving more than 50 MME per day, we found higher proportions of patients with Charlson co-morbidity scores greater than 1 (Table 2).

**Table 1:** Demographic characteristics and co-prescription of benzodiazepines and stimulants per average opioid dosage groups

	< 50 MME	50 to 90 MME	> = 90 MME	*p*-value
Female	442,574 (54.2%)	800 (45%)	477 (38%)	< 0.001
Age at 2010, mean (+ SD)	49.3 (+ 17.8)	52.2 (+ 17.3)	50.4 (+ 16.4)	< 0.001
Any benzodiazepine prescription	361,447 (44.2%)	1489 (83.7%)	1082 (86.1%)	< 0.001
Any stimulant prescription	26,157 (3.2%)	170 (9.6%)	172 (13.7%)	< 0.001
SES				
Very Low	51,404 (6.8%)	37 (2.2%)	24 (2%)	< 0.001
Low	226,346 (29.8%)	679 (41.2%)	473 (40%)	
Medium	255,960 (33.7%)	580 (35.2%)	424 (35.8%)	
High	173,825 (22.9%)	283 (17.2%)	213 (18%)	
Very High	52,959 (7%)	69 (4.2%)	49 (4.1%)	

*MME* morphine milliequivalents

**Table 2:** Distribution of Charlson co-morbidity score per MME dosage groups

Score	< 50 MME	50 to 90 MME	> = 90 MME	*p*-value
.00	304,282 (37.2%)	311 (17.5%)	208 (16.6%)	< 0.001
1.00	190,309 (23.3%)	351 (19.7%)	251 (20%)	
2.00	113,691 (13.9%)	324 (18.2%)	196 (15.6%)	
3.00	70,125 (8.6%)	222 (12.5%)	172 (13.7%)	
4.00	46,378 (5.7%)	184 (10.3%)	114 (9.1%)	
5.00	32,295 (4%)	107 (6%)	101 (8%)	
6.00	23,290 (2.9%)	98 (5.5%)	71 (5.7%)	
7.00	16,312 (2%)	73 (4.1%)	60 (4.8%)	
8.00	10,342 (1.3%)	50 (2.8%)	35 (2.8%)	
9.00	5729 (0.7%)	33 (1.9%)	27 (2.1%)	
10.00	2700 (0.3%)	12 (0.7%)	14 (1.1%)	
11.00	1118 (0.1%)	11 (0.6%)	5 (0.4%)	
12.00	396 (0%)	2 (0.1%)	2 (0.2%)	
13.00	102 (0%)	0 (0%)	0 (0%)	
14.00	22 (0%)	0 (0%)	0 (0%)	
15.00	5 (0%)	0 (0%)	0 (0%)	
> 17.00	1 (0%)	0 (0%)	0 (0%)	

**Fig. 1:**
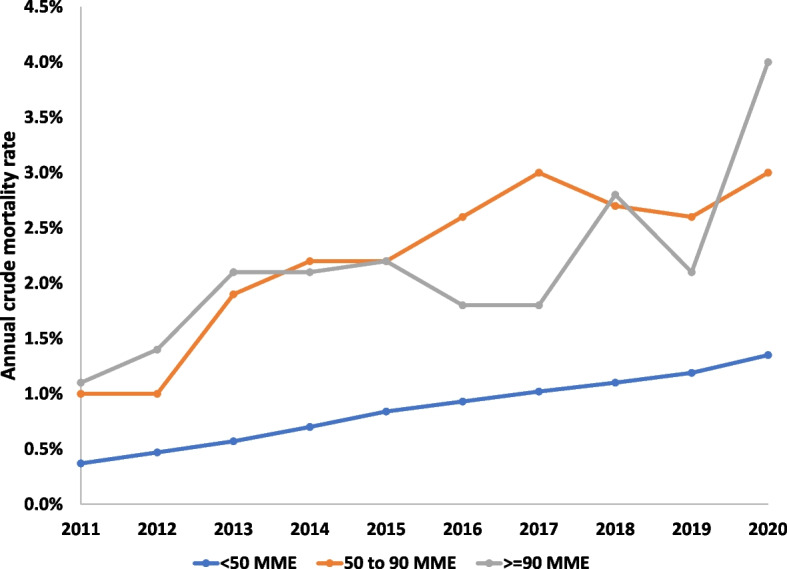
Annual mortality rates since 2011, among those alive on January 1st of each calendar year, per MME dosage group

During all years of follow-up, mortality was higher among patients receiving more than 50 MME per day (Fig. 1). On multivariate analysis, higher MME was associated with higher hazard ratios towards mortality (Table 3). Additionally, male sex, lower SES, and concomitant filling of benzodiazepine and stimulants were associated with increased mortality.

**Table 3:** Mortality hazard ratio (HR) estimates

	HR (95%CI)	*p*-value
MME		
< =50 MME	1	ref
50 MME up to 90 MME	2.23 (2.01 to 2.46)	< 0.001
> = 90 MME	2.37 (2.1 to 2.68)	< 0.001
Male vs Female	1.29 (1.27 to 1.31)	< 0.001
Age at 2010 (per year increase)	1.11 (1.10 to 1.12)	< 0.001
SES		
Very low	1	ref
Low	0.81 (0.78 to 0.84)	< 0.001
Medium	0.66 (0.63 to 0.68)	< 0.001
High	0.58 (0.55 to 0.6)	< 0.001
Very High	0.55 (0.52 to 0.57)	< 0.001
Any prescription of stimulant	1.23 (1.11 to 1.36)	< 0.001
Any prescription of benzodiazepine	1.18 (1.16 to 1.2)	< 0.001
Charlson score		
< =1	1	ref
2 or 3	1.76 (1.71 to 1.81)	< 0.001
4 or greater	3.57 (3.48 to 3.66)	< 0.001

Among patients aged 18 to 50 receiving more than 50 MME per day, the 10-year SMRs demonstrate increased mortality compared with the general population. This is evident among patients with a Charlson co-morbidity score of zero and persisted when examining patients with a score ranging from zero to 10 (Table 4).

**Table 4:** Standardized Mortality Ratios (95% confidence intervals) among patients aged 18 to 50 at 2010 with no oncologic diagnosis^a^

	Female		Male	
Charlson	50 to 90 MME	> = 90 MME	50 to 90 MME	> = 90 MME
0	6.52 (5.91–7.42)	10.6 (9.55–12.13)	1.2 (1.09–1.33)	2.68 (2.44–2.97)
0 to 1	6.86 (6.22–7.8)	8.64 (7.79–9.84)	2.44 (2.22–2.69)	3.35 (3.06–3.7)
0 to 2	5.8 (5.28–6.57)	8.07 (7.3–9.16)	2.76 (2.53–3.05)	3.23 (2.95–3.56)
0 to 3	5.38 (4.9–6.08)	8.07 (7.32–9.15)	3.23 (2.96–3.56)	3.64 (3.33–4)
0 to 4	6.04 (5.51–6.82)	8.81 (7.99–10)	3.22 (2.95–3.54)	3.99 (3.66–4.39)
0 to 5	6.6 (6.02–7.45)	8.52 (7.72–9.66)	3.42 (3.13–3.75)	4.56 (4.18–5.01)
0 to 6	7.61 (6.95–8.6)	8.93 (8.1–10.12)	3.37 (3.09–3.71)	4.54 (4.16–4.98)
0 to 7	7.55 (6.9–8.52)	9.17 (8.33–10.39)	3.65 (3.35–4.01)	4.94 (4.53–5.42)
0 to 8	8.24 (7.53–9.31)	9.08 (8.25–10.28)	3.8 (3.49–4.18)	5.22 (4.79–5.73)
0 to 9	8.56 (7.82–9.66)	9.08 (8.25–10.28)	3.76 (3.45–4.13)	5.16 (4.74–5.67)
0 to 10	8.56 (7.82–9.66)	8.98 (8.16–10.16)	3.76 (3.45–4.13)	5.38 (4.94–5.91)

^a^Standard population in the entire Israeli population aged 18 to 50

## Discussion

We found that increased use of opioids is associated with increased all-cause mortality among non-oncology patients. Throughout the years 2011 to 2020, the annual crude proportion of patients that died was higher among those filling more than 50 MME per day. The multivariate model, controlling for demographic features and comorbidities, demonstrated increased hazard ratios for increased mortality with higher dosages of filled opioid prescriptions. Finally, we present indirectly standardized mortality ratios, among young healthy adults, in which we found that the observed mortality was higher than expected, based on national Israeli life-tables published by the Central Bureau of Statistics. These results, we believe, satisfy our hypothesis, that there exists in Israel an independent association between opioid use and increased mortality.

This is the first report to show that increased use of opioids, in Israel, is associated with increased all-cause mortality among non-oncologic patients. Previous Israeli studies have examined this question through death certificates that are based on medical records or laboratory tests indicating opioid use [2, 13]. In Israel, the toxicology screen assay used in all hospitals and medical examiner facilities does not detect fentanyl or oxycodone, or any of their derivatives [23]. Previous Israeli studies did not undertake large population-based analyses comparing mortality in opioid dosage groups. We believe that the previous reports might have underreported true opioid-associated mortality due to missed diagnoses and incorrect classification in the available medical records. Feingold et al. do not discuss their methods of confirmation of the cause of death as a potential limitation but rather provide other reasons for their findings, such as increased use of medical marijuana and less frequent use of oral opioid substances [13]. Shapira et al. also rely solely on death certificate reports and they provide no explanation for their findings of stable low opioid-related death rates [2]. We should note that the association we found does not prove that opioid use was the direct cause of death, but rather that filled prescriptions of higher dosages were associated with higher mortality.

Patient comorbidities and non-opioid associated risk of death could potentially confound the association we sought to examine. Increased comorbidities are associated with increased mortality and, as shown in Table 2, we found an association between higher dosages of opioids and comorbidities. To control and assess the independent association of opioid dosage and mortality, we excluded oncologic patients, examined the associations in a multivariate model (controlling also for another potential confounder – socio-economic status) and generated indirectly standardized mortality ratios per comorbidity group.

Comparing our findings with those published in Europe and in North America, the crude number of mortality events in Israel seems far smaller among patients who filled more than 50 MME per day [8, 10, 24]. There were 737 deaths among these patients, and though they were non-oncology patients, some harbored significant co-morbidities. The small absolute number of deaths, coupled with the higher-than-expected mortality rates, most notably among young and healthy adults, suggest that our findings represent only the beginning of a far greater mortality burden that may become evident in the coming years. This should cause policy leaders to appreciate that the indication of harm has become evident and will likely increase over time.

It is known that prescription fentanyl can be found in illegal secondary markets in Israel [2, 23, 25]. However, increased mortality could just as well have resulted from association with criminal elements, even though the authors think this explanation cannot be the sole reason for increased mortality. It should be recognized that our analysis would not include mortality among people who used prescription opioids that were obtained through illegal secondary markets. Cases such as these would cause an underestimation of true opioid-associated all-cause mortality from all users, not only those who themselves were prescribed the opioids that are included in this analysis. We should also consider that opioids act in several mechanisms that can shorten the lifespan [26], rather than solely causing death through overdoses.

This report and its derived conclusions are not without limitations which should be stated and addressed. We include only patients with prescriptions filled within the public health system in community pharmacies. We have no data regarding private pharmacy prescription filling or opioids provided within hospital and emergency medicine settings. Similar to other reports, we included all patients who had at least one prescription during the study period. This means that both acute and chronic treatments are collected and that there is no differentiation between patients who received small doses over long periods and patients who received high doses over short periods. To cope with this matter we summed, per patient, all MMEs of filled prescriptions, from the first prescription until censoring or death, and divided by length of follow-up. In this manner, we identified those patients who, cumulatively, in one way or another, received larger quantities of opioid medications. Certainly, these are crude estimates that were used for this analysis and are not necessarily useful for surveillance and policy. Finally, it is worth noting that the opioid prescribing decision of clinicians is often more nuanced between different types of opioids and within the opioid MME categories examined in this report.

During the study period, there was a consistent increase in annual mortality rates in all age groups. This likely is the consequence of the study cohort growing older, and a gradual increase in the proportion of older adults in the study population.

In CHS, aside from buprenorphine patches, the medication in its oral form is not part of the pharmacology formulary. Patients provided buprenorphine in Ministry of Health addiction clinics are not provided the medication through CHS. The study presented includes only CHS patients. To our knowledge, there are a handful of addiction clinics embedded within other Israeli HMOs that do provide and prescribe buprenorphine as an oral medication and addiction substitute.

Policy seeking to curtail the increased and potentially not medically-indicated use of opioids can be implemented in various ways. Promoting wide-scope prohibitions on opioid prescriptions may drive patients requiring or otherwise desiring to use opioids to the illegal secondary market, as has been documented in the past in the USA [12, 27]. Conversely, more focused and directed schemes, aiming to curb specific prescription patterns and use, might be effective at limiting non-medically-indicated opioid use.

Until 2022, representatives of the Israeli Ministry of Health (IMOH) sporadically inspected the four HMOs in a non-standardized process with included site visits and interviews with medical staff and HMO administrations. These actions seem to have been fruitless. In September 2022 the IMOH released a directive announcing a renewed interest in preventing opioid prescription misuse and opioid addiction. Among its recommendations are the establishment of standing committees and opioid control policies in every health institution (hospitals and HMOs), continued collection of data concerning opioid use, and sharing these with the IMOH and other health providers, including addiction professionals. This process aims to provide educational resources and an empiric knowledge repository assisting rational policy. The IMOH further announced its intention to limit maximum daily doses of fentanyl for non-oncologic patients, limit the use of hand-written opioid prescriptions, and require private pharmacies to report all of their opioid dispensing. As of yet, This new policy has not been fully implemented since parliament approval of these measures is still pending. CHS has, since July 2022, introduced an authorization process for the preparation of any fentanyl prescription. This intervention has led to a decrease in the number of patients receiving fentanyl as well as a decrease in the total amount of fentanyl procured by its HMO members (data not published).

## Conclusions

This is the first study to demonstrate that also in Israel, in contrast to previous reports, there is an association between increased opioid prescription filling and increased mortality, notably among the younger the less morbid patients. The challenges posed by these findings echo those encountered in other countries. The dangers of inaction are evident and are expected to lay a significant toll on the Israeli public’s health. Healthcare regulators and providers should, therefore, act to curtail the increasing opioid prescriptions and introduce controls and limitations in the system, which, currently, has no such mechanisms in place.

## Data Availability

Data not available.

## References

[1] Davidovitch N, Kranzler Y, Miron O (2023). Are we nearing an opioid epidemic in Israel?.

[2] Shapira B, Berkovitz R, Haklai Z, Goldberger N, Lipshitz I, Rosca P (2023). Trends and correlated outcomes in population-level prescription opioid and transdermal fentanyl use in Israel. Isr J Health Policy Res..

[3] Ponizovsky AM, Marom E, Weizman A, Schwartzberg E (2018). Changes in consumption of opioid analgesics in Israel 2009 to 2016: an update focusing on oxycodone and fentanyl formulations. Pharmacoepidemiol Drug Saf..

[4] Dressler RL, Kaliner E, Cohen MJ (2023). Trends in Israeli community-based opioid prescribing, 2010-2020, an observational study of the country's largest HMO. Isr J Health Policy Res..

[5] Curtis HJ, Croker R, Walker AJ, Richards GC, Quinlan J, Goldacre B (2019). Opioid prescribing trends and geographical variation in England, 1998-2018: a retrospective database study. Lancet Psychiatry..

[6] Fischer B, Jones W, Urbanoski K, Skinner R, Rehm J (2014). Correlations between prescription opioid analgesic dispensing levels and related mortality and morbidity in Ontario, Canada, 2005-2011. Drug Alcohol Rev..

[7] Guy GP, Zhang K, Bohm MK, Losby J, Lewis B, Young R (2017). Vital signs: changes in opioid prescribing in the United States, 2006-2015. MMWR Morb Mortal Wkly Rep..

[8] Pierce M, van Amsterdam J, Kalkman GA, Schellekens A, van den Brink W (2021). Is Europe facing an opioid crisis like the United States? An analysis of opioid use and related adverse effects in 19 European countries between 2010 and 2018. Eur Psychiatry..

[9] Miron O, Zeltzer D, Shir T, Balicer RD, Einav L, Feldman BS (2020). Rising opioid prescription fulfillment among non-cancer and non-elderly patients-Israel's alarming example. Reg Anesth Pain Med..

[10] Gomes T, Tadrous M, Mamdani MM, Paterson JM, Juurlink DN (2019). The burden of opioid-related mortality in the United States. JAMA Netw Open..

[11] Kariisa M, Davis NL, Kumar S, Seth P, Mattson CL, Chowdhury F, Jones CM (2022). Vital signs: drug overdose deaths, by selected sociodemographic and social determinants of health characteristics - 25 states and the District of Columbia, 2019-2020. MMWR Morb Mortal Wkly Rep..

[12] Tanz LJ, Dinwiddie AT, Mattson CL, O'Donnell J, Davis NL (2022). Drug overdose deaths among persons aged 10-19 years - United States, July 2019-December 2021. MMWR Morb Mortal Wkly Rep..

[13] Feingold D, Goldberger N, Haklai Z, Lev-Ran S (2017). Fatal overdoses of opioids in Israel 2005-2014. Eur Addict Res..

[14] Roshka P, Spivak P, Goldman K, Austin A (2019). Summary of the activities of the Department for the Treatment of addictions - mental health division.

[15] Charlson ME, Pompei P, Ales KL, MacKenzie CR (1987). A new method of classifying prognostic comorbidity in longitudinal studies: development and validation. J Chronic Dis..

[16] Hernandez-Avila M, Ortiz-Brizuela E, Tamayo-Ortiz M, Zepeda-Tello R, Gutierrez-Diaz H, Barros-Sierra Cordera D (2023). Assessing the real-world effectiveness of five SARS-CoV-2 vaccines in a cohort of Mexican pensioners: a nationwide nested test-negative design study. Lancet Reg Health Am..

[17] Onyewuenyi TL, Peterman K, Zaritsky E, Ritterman Weintraub ML, Pettway BL, Quesenberry CP (2023). Neighborhood disadvantage, race and ethnicity, and postpartum depression. JAMA Netw Open..

[18] Swarbrick C, Poulton T, Martin P, Partridge J, Moppett IK, Team SP (2023). Study protocol for a national observational cohort investigating frailty, delirium and multimorbidity in older surgical patients: the third Sprint National Anaesthesia Project (SNAP 3). BMJ Open..

[19] Israeli, Social, Security. Membership in Health Maintenance Organizations - Synopsis - Israeli Social Security 2023 [Available from: https://www.btl.gov.il/Mediniyut/Situation/haveruth1/Pages/default.aspx.

[20] Von Korff M, Saunders K, Thomas Ray G, Boudreau D, Campbell C, Merrill J (2008). De facto long-term opioid therapy for noncancer pain. Clin J Pain..

[21] Dowell D, Haegerich TM, Chou R (2016). CDC Guideline for Prescribing Opioids for Chronic Pain--United States. JAMA..

[22] Israeli, Central, Bureau, of, Statistics. [Available from: https://www.cbs.gov.il/en/publications/Pages/2022/Complete-Life-Tables-of-Israel-2016-2020.aspx.

[23] Greenberg N. Addiction to Fentanyl Patches in Israel - background data for parliamentary committee: Israeli Parliment - The Knesset; 2023 [Available from: https://main.knesset.gov.il/activity/info/research/pages/incident.aspx?rid=7579&businesstype=1.

[24] Fischer B (2023). The continuous opioid death crisis in Canada: changing characteristics and implications for path options forward. Lancet Reg Health Am..

[25] Shapira B, Rosca P, Berkovitz R, Gorjaltsan I, Neumark Y (2020). The switch from one substance-of-abuse to another: illicit drug substitution behaviors in a sample of high-risk drug users. PeerJ..

[26] Kotlinska-Lemieszek A, Zylicz Z (2022). Less well-known consequences of the long-term use of opioid analgesics: a comprehensive literature review. Drug Des Devel Ther..

[27] Wilson N, Kariisa M, Seth P, Ht S, Davis NL (2020). Drug and opioid-involved overdose deaths - United States, 2017-2018. MMWR Morb Mortal Wkly Rep..

